# Heterogeneous Ag-TiO_2_-SiO_2_ composite materials as novel catalytic systems for selective epoxidation of cyclohexene by H_2_O_2_

**DOI:** 10.1371/journal.pone.0176332

**Published:** 2017-05-11

**Authors:** Xin Wang, Jianyue Xue, Xinyun Wang, Xiaoheng Liu

**Affiliations:** 1 College of Chemistry and Materials Engineering, Chaohu University, Chaohu, Anhui, China; 2 Institute of Novel Functional Materials and Fine Chemicals, Chaohu University, Chaohu, Anhui, China; 3 Key Laboratory of Soft Chemistry and Functional Materials of the Ministry of Education, Nanjing University of Science and Technology, Nanjing, Jiangsu, China; Argonne National Laboratory, UNITED STATES

## Abstract

TiO_2_-SiO_2_ composites were synthesized using cetyl trimethyl ammonium bromide (CTAB) as the structure directing template. Self-assembly hexadecyltrimethyl- ammonium bromide TiO_2_-SiO_2_/(CTAB) were soaked into silver nitrate (AgNO_3_) aqueous solution. The Ag-TiO_2_-SiO_2_(Ag-TS) composite were prepared via a precipitation of AgBr in soaking process and its decomposition at calcination stage. Structural characterization of the materials was carried out by various techniques including X-ray diffraction (XRD), scanning electron microscopy (SEM), transmission electron microscopy (TEM), N_2_ adsorption-desorption and ultraviolet visible spectroscopy (UV-Vis). Characterization results revealed that Ag particles were incorporated into hierarchical TiO_2_-SiO_2_ without significantly affecting the structures of the supports. Further heating-treatment at 723 K was more favorable for enhancing the stability of the Ag-TS composite. The cyclohexene oxide was the major product in the epoxidation using H_2_O_2_ as the oxidant over the Ag-TS catalysts. Besides, the optimum catalytic activity and stability of Ag-TS catalysts were obtained under operational conditions of calcined at 723 K for 2 h, reaction time of 120 min, reaction temperature of 353 K, catalyst amount of 80 mg, aqueous H_2_O_2_ (30 wt.%) as oxidant and chloroform as solvent. High catalytic activity with conversion rate up to 99.2% of cyclohexene oxide could be obtainable in water-bathing. The catalyst was found to be stable and could be reused three times without significant loss of catalytic activity under the optimized reaction conditions.

## Introduction

The direct oxidation of hydrocarbons is a field of academic and industrial importance [1,2]. From an industrial point of view, epoxides are largely used for the synthesis of perfume materials, anthelmintic preparations, epoxy resins, plasticizers, drugs, sweeteners, etc [3]. Selective oxidation of olefins to epoxides is a pivotal reaction in trade mark of fine chemical synthesis [4]. The oxidants for the epoxidation of alkenes include peracids, organic peroxides, hydrogen peroxide, and molecular oxygen [5]. Epoxidation reactions of alkenes generally require the presence of a catalyst. Compared with homogeneous catalysts, heterogeneous catalysts have many advantages such as easy separation and facile recovery of the solid catalyst from the reaction mixture for recycling without tedious work up [6]. Heterogeneous catalytic systems are better than similar homogenous catalytic systems in terms of separation of reaction products from the catalyst [7,8]. Up to now, transition metals or rare earth modified metal oxides have been studied widely [9,10]. Noble metals (such as Ag) have also received extensive attention due to their important potential applications [11,12]. However, noble metal modified composite oxide on the special interface have rarely been investigated. Therefore, the epoxidation of alkene derivatives using Ag as heterogeneous catalyst has been studied [13]. Cyclohexene oxide and its epoxide are important intermediate in organic process industry, and can be made by epoxidation of cyclohexene [14,15]. Therefore the catalytic epoxidation of cyclohexene remains challenging in industry. The catalytic epoxidation using metal oxides as catalysts with high selectivity under mild reaction conditions has become an important researching field [16,17]. The development of catalysts that may operate at room temperature and pressure for the transformation of relatively cheap and available substrates into valuable functionalized products has attracted the attention from both academy and industry [18]. Recently, many researchers have designed a series of strategies to load noble metal (Ag) for the catalytic reaction [19–21].

In this work, Ag-TS composite were prepared through a self-assembly process by soaking TiO_2_-SiO_2_ in AgNO_3_ aqueous, and the influence of catalytic activity of Ag-TS composite were also discussed. Their catalytic performances for the oxidation of cyclohexene using H_2_O_2_ as oxidant were studied, and the effects of Ag content in the catalysts were investigated. Compared with the above reported methods, this process does not need any additional reagents. The calcined composite materials were used as the catalyst for the epoxidation of cyclohexene.

## Materials and methods

### Preparation of Ag-TiO_2_-SiO_2_ composite

All the chemicals were obtained from Nanjing Chemical Reagents and used without further purification. Deionized water was used in all sample preparation. Titanium(IV)n-butoxide (0.7 g) and TEOS(0.5g) were added dropwise to a TEA (6.5 g) solution. The mixture was stirred for 25 min at 298 K until a pale yellow solution was obtained, which was then transferred to a sample trough. Toward this, 0.15 g of gelatin was dissolved in 23 g of deionized water and 0.6 g of CTAB with stirring at 303 K. The sample though was sealed and left undisturbed at 293–297 K for 48 h until a film to present on its air-water interface. The film was transferred to a glass substrate, washed by deionized water to remove excess CTAB, and then soaked in a silver nitrate (AgNO_3_) aqueous solution of 0.05 mol·L^-1^ for 48 h. Finally, the soaked film was heated in a muffle at different temperature for 2 h.

### Characterizations

The X-ray diffraction patterns of samples were obtained at Rigaku D/max2500 X-ray diffractometer (XRD) using Cu Kα radiation (λ = 0.154 nm). Scanning electron microscope (SEM) of JEOL-6380 LV and energy dispersive spectrometer (EDS) with EDAX Genesis 2000 were applied to determine the morphologies and compositions. The morphologies were further identified with a JEOL JEM-2100 transmission electron microscope (TEM) at 200 kV coupled with a Gatan794 charge coupled device (CCD) camera, N_2_ adsorption-desorption measurements were performed on a Micromeritics ASAP 2020 apparatus. All samples were ultrasonically dispersed in water and dried over a copper grid. The ultraviolet visible (UV-Vis) spectra were recorded on a Beijing Persee UV-T9CS spectrometer in the wavelength range of 300–600 nm at room temperature.

### Epoxidation of cyclohexene

The catalytic performance of Ag-TS composite for the epoxidation of cyclohexene was investigated in a 100 mL erlenmeyer flask equipped with a stirrer, thermometer, and reflux condenser. In a typical batch experiment, to a flask containing 80 mg of catalyst, 12 mL of chloroform and 6 mL of 30% mass fraction H_2_O_2_ were added into the flask. Subsequently, cyclohexene (6 mL) was added and the contents of the flask were kept in the water bath on a magnetic hot plate for 120 min with continuous stirring at 353 K. After reaction for 2 h, the catalyst was separated from the reaction solution by centrifugation. Then this separated catalyst was dried at 353 K overnight and calcined in air at 723 K for 120 min, to obtain the regenerated catalyst. The catalytic performance of the regenerated catalyst was investigated as the same procedure as the fresh catalyst. The concentrations in the solution was analyzed by High Performance Liquid Chromatography (HPLC) of DIONEX U-3000 with an ultraviolet detector of VWD-3100. The chromatographic conditions were as following: 0.02 mol·L^-1^ methanol aqueous solution was used as mobile phase, the flow rate was set at 1.0 mL·min^-1^, the wavelength of UV irradiation was 210 nm, the column temperature was 298 K and the injection volume was 10 μL. The epoxide yield, the selectivity, the conversion of cyclohexene oxide and the selectivity to by-products were evaluated using Eqs (1–4):

$$Expoxide\;yield=(n_{epoxide}/n_{cyclo-C_{6}H_{10},\mathrm{int}roduced})\times 100$$ (1)

$$Selectivity\;to\;epoxide=(n_{epoxide}/n_{cyclo-C_{6}H_{10},\mathrm{int}roduced}-n_{cyclo-C_{6}H_{10},unreacted})\times 100$$ (2)

$$Conversion\;of\;cyclo-C_{6}H_{10}=(n_{cyclo-C_{6}H_{10},\mathrm{int}roduced}-n_{cyclo-C_{6}H_{10},unreacted})/n_{cyclo-C_{6}H_{10},\mathrm{int}roduced}\times 100$$ (3)

$$Selectivity\;\;to\;by-products=n_{by-product,i}/(n_{cyclo-C_{6}H_{10},\mathrm{int}roduced}-n_{cyclo-C_{6}H_{10},unreacted})\times 100$$ (4)

## Results and discussion

### Morphology and structure

**Fig 1:**
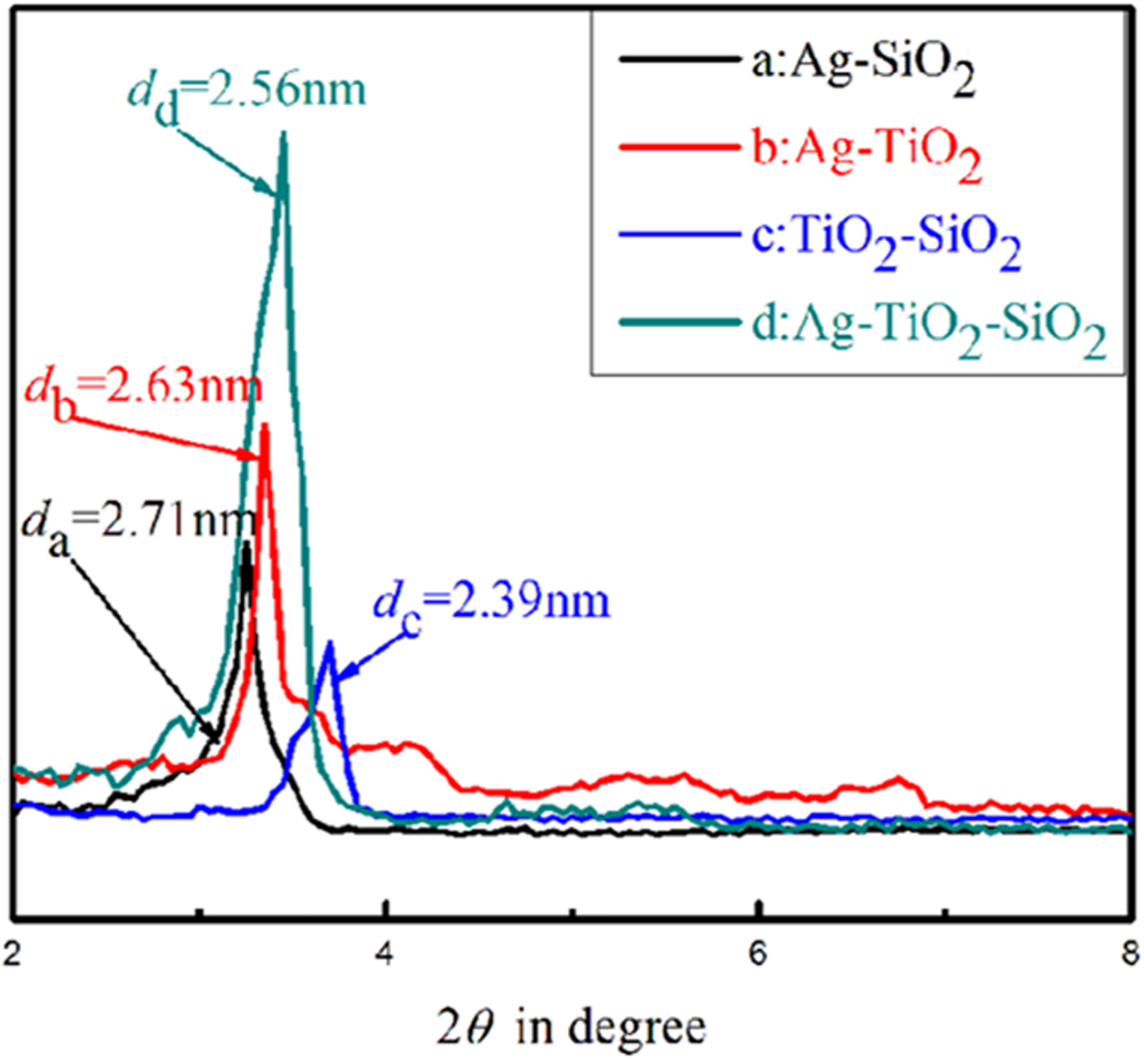
LAXRD patterns of TiO_2_-SiO_2_ and Ag-TiO_2_-SiO_2._

The XRD pattern of the four uncalcined materials are displayed in Fig 1 (a:Ag-SiO_2_; b:Ag-TiO_2_; c:TiO_2_-SiO_2_; d:Ag-TiO_2_-SiO_2_). A predominant peak could be observed with four samples in the 2*θ* range from 2° to 8°. This obvious reflection appeared at small angle region which implied ordered structures inside of the self-assembled inorganic-organic metal oxide [22,23]. This suggests that as-prepared TiO_2_-SiO_2_ materials are amorphous product. Moreover, the *d* value corresponded to the strongest peak meant for the spacing between the ordered lamellas or the diameter of the ordered mesopores inside the synthesized composite materials. The predominant peak of TiO_2_-SiO_2_ (c) at 2*θ* = 3.75° give a d value of 2.39 nm. Comparison, the diffraction peak of TiO_2_-SiO_2_ product is much weaker than that of Ag-doped different materials and it is obvious that the 2*θ* angles of Ag-doped products moved toward the smaller values, indicating the increased layer-layer or pore-pore correlation distance with an increase of Ag content.

**Fig 2:**
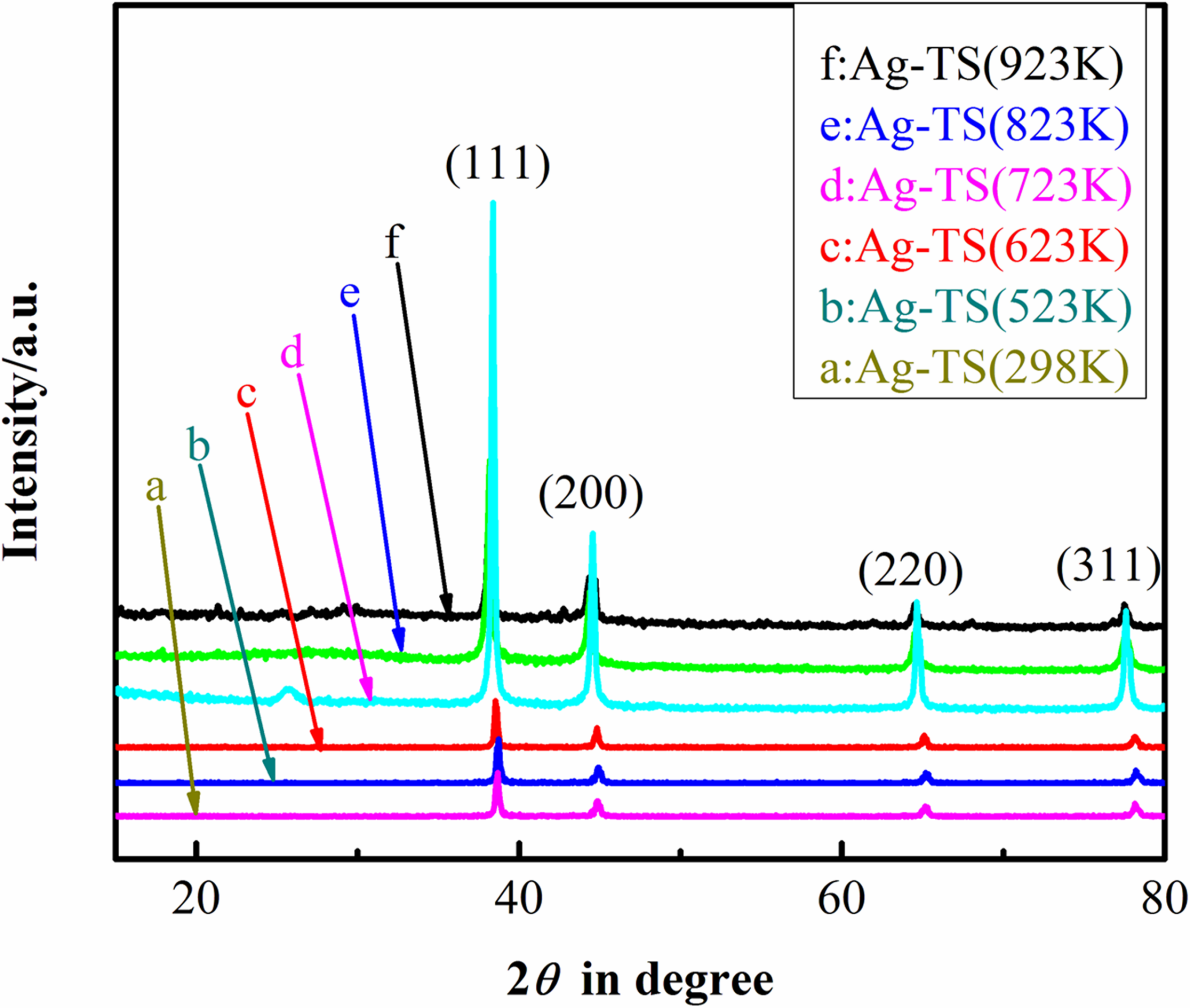
HAXRD patterns of Ag-TS composite before and after calcination at different temperature.

The large-angle XRD patterns of the Ag-TS materials before and after calcination at different temperature are displayed in Fig 2. Those samples obtain the same diffraction peaks, indicates that these samples contain the same chemical composition. These peaks observed at 2*θ* values of 38.3, 44.2, 64.5 and 77.4° are assigned to (111), (200), (220) and (311) lattice planes of fcc metallic Ag (JCPDS Card NO. 04–0784), respectively. The lattice parameter calculated from the XRD pattern is 0.4078 nm, in agreement with the literature report [24]. It can be seen that the diffraction peaks (d) is very strong, indication of good crystallinity of the Ag-TS composite grain after calcination at 723 K. It shows that calcination does not change the crystalline phase of the Ag particles. The Ag-doped to the TiO_2_-SiO_2_ particles is characteristic crystalline form, 2*θ* values of the major peaks locate in the range from 15° to 80° in accordance with the characteristic diffraction of Ag verifying.

**Fig 3:**
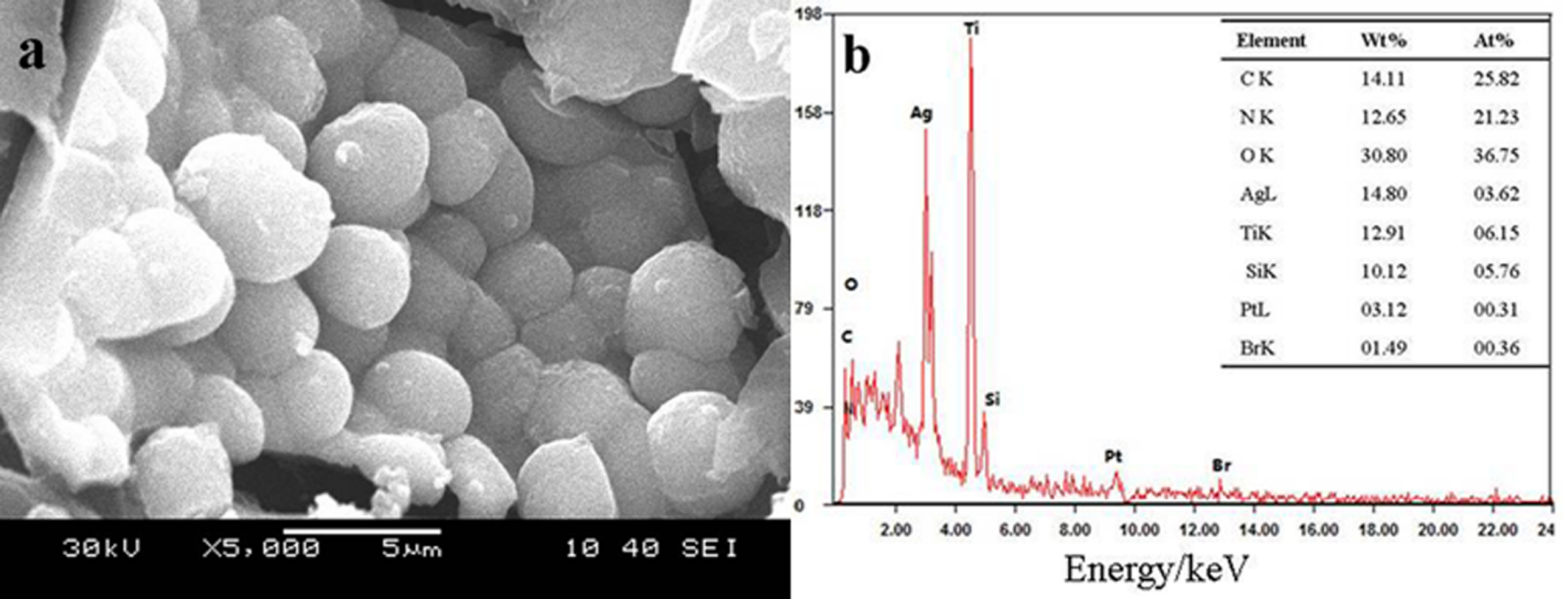
SEM image and EDS spectra of Ag-TS composite after calcination at 723K.

The SEM micrographs coupled with Energy Disperse Spectroscopy (EDS) analysis are shown in Fig 3. This shows the SEM image(a) and EDS spectrum(b) of the Ag-TS materials calcinated at 723 K. The SEM image in Fig 3A shows the Ag-TS composite have particle diameter ranging from 300 nm to 500 nm and the surface of the particles is rough. They are composed of many small spherical aggregations. The EDS spectrum confirms the presence of Ag, Ti, Si and O in the composite, and the rest could be identified as C, N and Pt that originated from the tape and sputtering source. The Pt signals come from the plated element.

**Fig 4:**
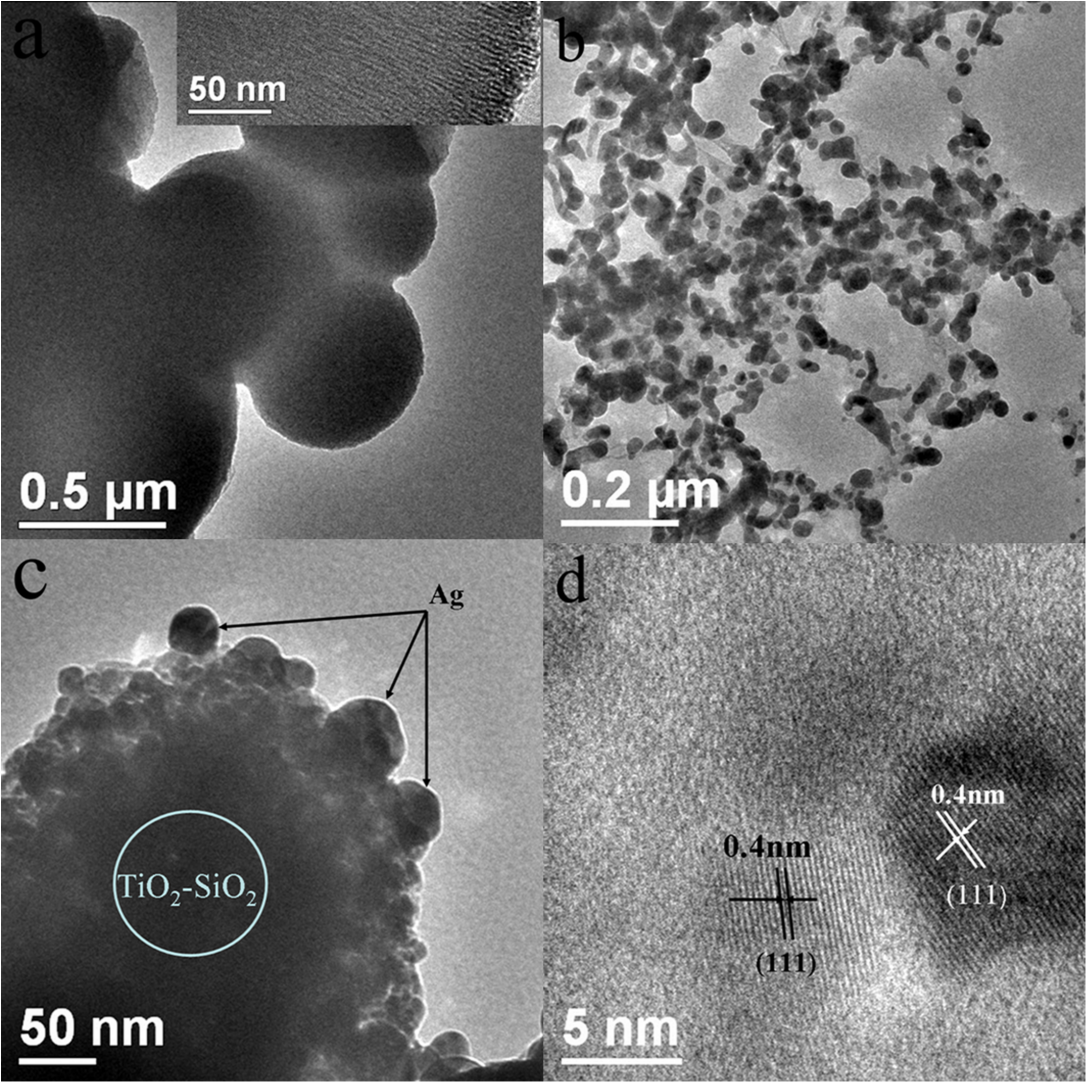
TEM images after calcination at 723 K of (a) TiO_2_-SiO_2_, (b) Ag (c) Ag-TS and (d)HRTEM image of Ag-TS.

For the sake of comparison, TEM images(Fig 4) of the three different product are examined. Fig 4A shows that the granularity of spherical TiO_2_-SiO_2_ particles spread at interlayer spacing of 300–600 nm. The appearance of TiO_2_-SiO_2_ in the inset TEM image in Fig 4A indicates the formation of structure, the hierarchical structure of the TiO_2_-SiO_2_ particles is consistent with the XRD results. It can be seen that all the Ag particles have similar spherical form in Fig 4B. The Ag particles have a range of 30–40 nm with broad size distribution. Fig 4C shows the Ag particles attached to surface of TiO_2_-SiO_2_ particles tend to grow 50–60 nm and the shape of Ag-TS particles appeared conglomerated. The Ag particles linked to TiO_2_-SiO_2_ are unambiguously, further reveals the composite materials of the structure. High resolution TEM image(Fig 4D) further confirms the presence of crystalline Ag nanodomains with a lattice spacing of 0.4 nm on the products. This clearly demonstrates that Ag particles were formed and firmly doped on the TiO_2_-SiO_2_ particles.

**Fig 5:**
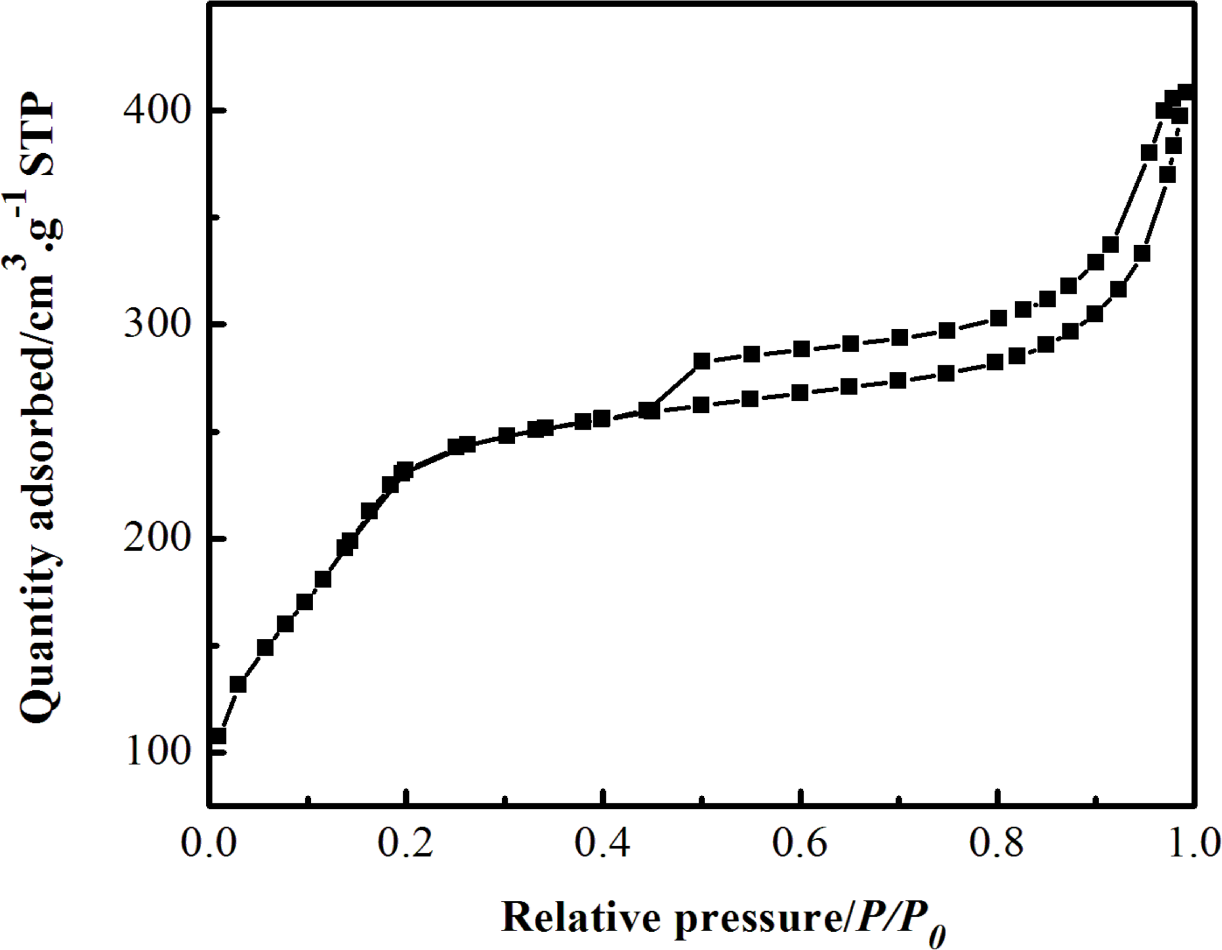
N_2_ adsorptionedesorption isotherms of Ag-TS at 77 K.

The specific surface area of Ag-TS composite can be determined by the N_2_ adsorption-desorption measurements. In Fig 5, Ag-TS displays a type IV adsorption- desorption isotherms for mesoporous structure according to the IUPAC classification [25]. Furthermore, the type H4 hysteresis loop suggests that the slit-like mesopores formed by the Ag-TS agglomerate, which is in line with the TEM observation(Fig 4C). The Brunauer-Emmett-Teller (BET) surface area and pore volume are 918.9 m^2^·g^-1^ and 0.57 cm^3^·g^-1^, respectively, based on the desorption branch.

**Fig 6:**
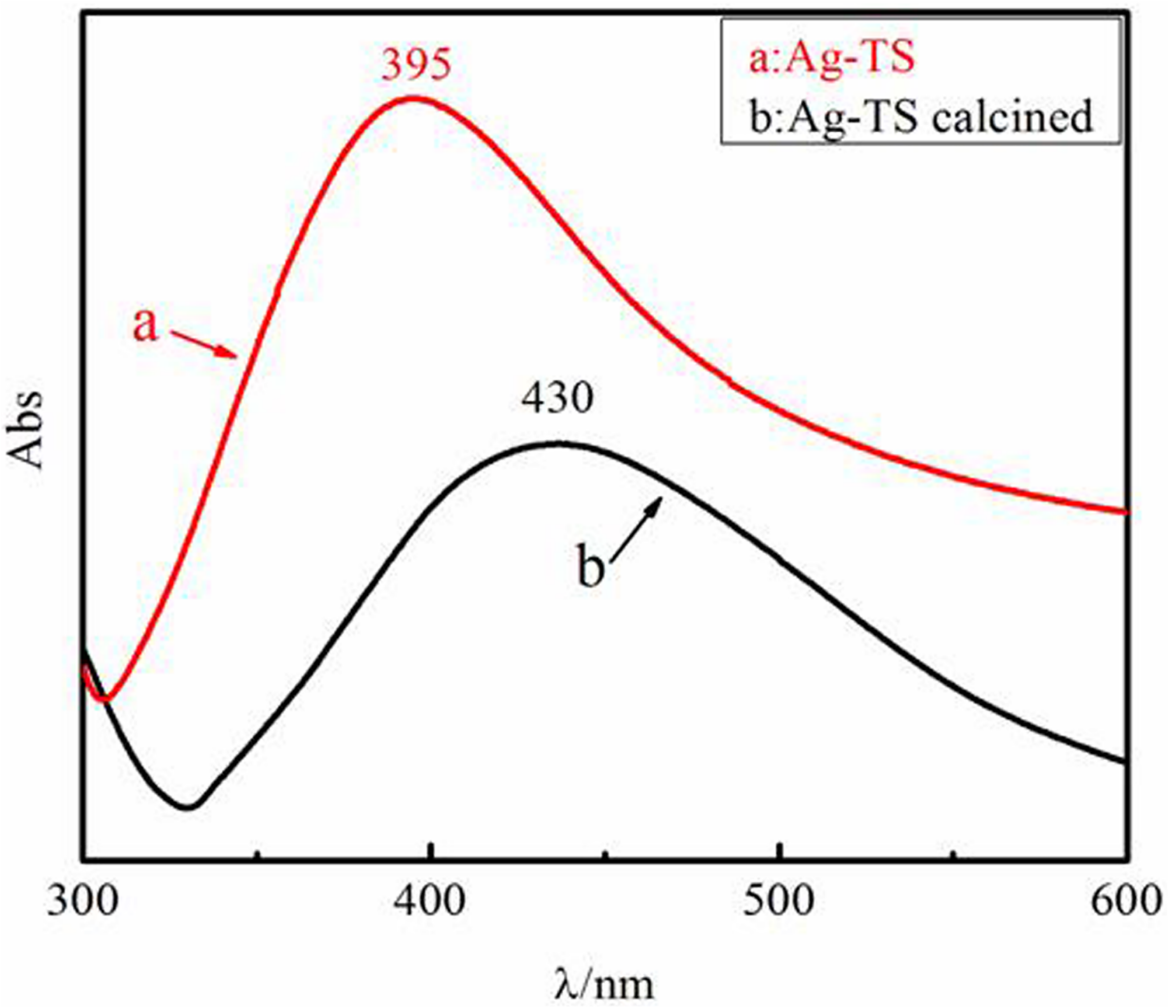
UV-Vis spectra of (a) Ag-TS particles; (b) Ag-TS particles after calcination at 723 K.

The UV-Vis spectra of both pristine and calcined Ag-TS composite are presented in Fig 6. TiO_2_-SiO_2_ do not show any UV visible absorption in the visible range. No absorption in the visible range for both samples could be observed while there is a strong absorption for Ag particles at *λ* of 390–430 nm. Compared with uncalcined sample with surface plasmon resonance peak at 395 nm, the same peak of calcined sample is red-shifted to 430 nm and broadened. The shift can be interpreted as the result of nano-size effect [26].

### Formation mechanism

**Fig 7:**
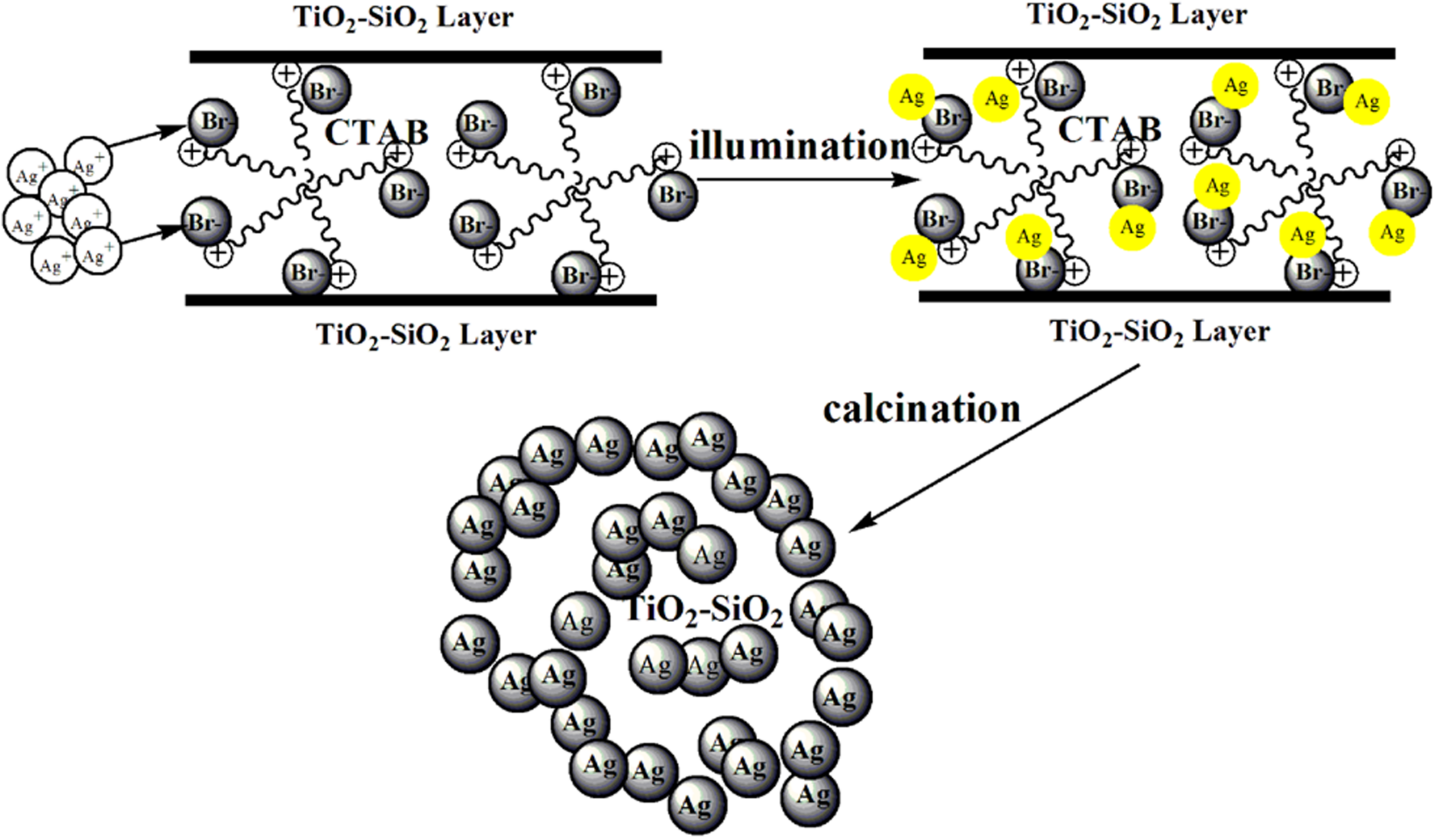
Formation mechanism of the Ag-TS composite.

The proposed formation mechanism of Ag-TS composite is illustrated in Fig 7. Initially, several CTAB molecules form a spherical micelle [27], the TiO_2_-SiO_2_ particles with negative charges interact with the positively charged CTAB micelle through electrostatic interactions. This template plays a key role in the subsequent assembling processes [28]. When TiO_2_-SiO_2_ particles formed on the air-water interfacial are soaked into the AgNO_3_ aqueous solution, the AgBr-TiO_2_-SiO_2_ composite are formed due to the precipitation of AgBr at the surfaces and voids of TiO_2_-SiO_2_ particles. The AgBr-TS particles can be prepared by a reaction of Ag ions and Br ions from CTAB. Then AgBr decomposes into elemental Ag particles under visible light irradiation. The surfactant(CTAB) is destructed and it has formed a stable structure of composite particles during the calcination process. Part of the Ag particles are attached to the surface, and the rest particles remain in the interior of the TS particles.

### Epoxidation of cyclohexene

**Fig 8:**
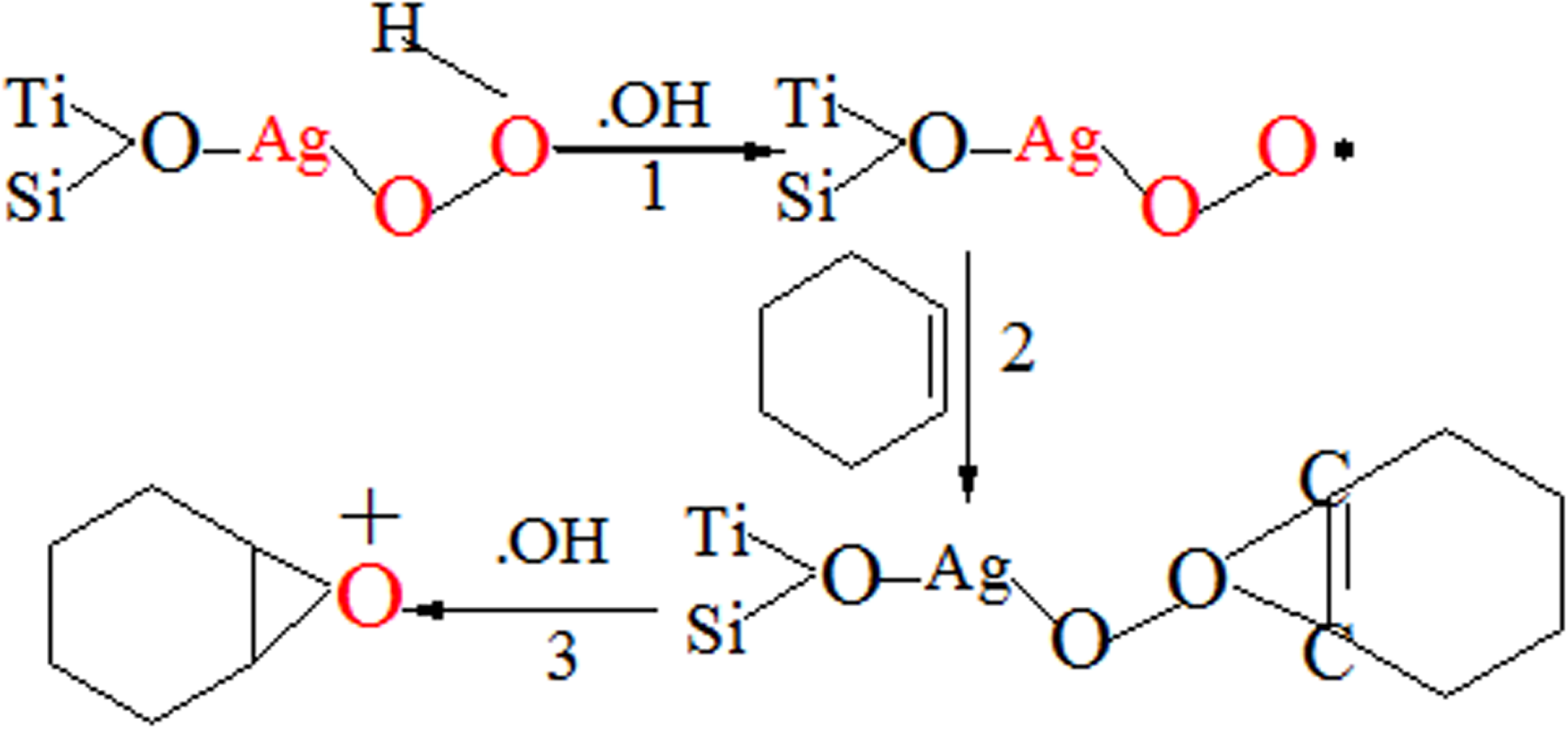
Plausible mechanism of epoxidation reaction.

It is generally accepted that the first step in the epoxidation by the Ag- TiO_2_-SiO_2_/H_2_O_2_ system is the formation of the hydroperoxo species, which are Ag particles here. The epoxidation mechanism of cyclohexene over calcined Ag-TS composite is briefly depicted in Fig 8. The catalytic reaction is similar to that occurred over TS-1 zaolite [29]. Firstly, the Ag particles react with H_2_O_2_ to form AgOOH [30]. Then AgOOH reacts with the OH· radical(the decomposition of H_2_O_2_) to form the Ag-peroxo radicals species [31]. This radical interacts with C = C double bond of the cyclohexene to form a new organic intermediate. Finally, the OH· radical terminates the reaction and forms the cyclohexene epoxide. AgOOH is recovered and thus the catalytic cycle could be maintained [32] and cyclohexene epoxide is the major product of cyclohexene oxidation. On the other hand, oxidation proceeds when H_2_O_2_ is activated on adjacent Ag sites, whereas hydrolysis to cyclohexanol occurs in the case that H_2_O adsorb on acidic OH· radical. From the fact that the former dominated the latter, even in the reaction with 30 wt % H_2_O_2_ aqueous solution, the activation of H_2_O_2_ is regarded as more frequent than H_2_O, and the activated peroxo species are more reactive than H_3_O^+^ species. In this mechanism, the absence of detection of cyclohexene oxide is attributed to the rapid hydrolysis catalyzed by acidic OH· radical, too.

**Fig 9:**
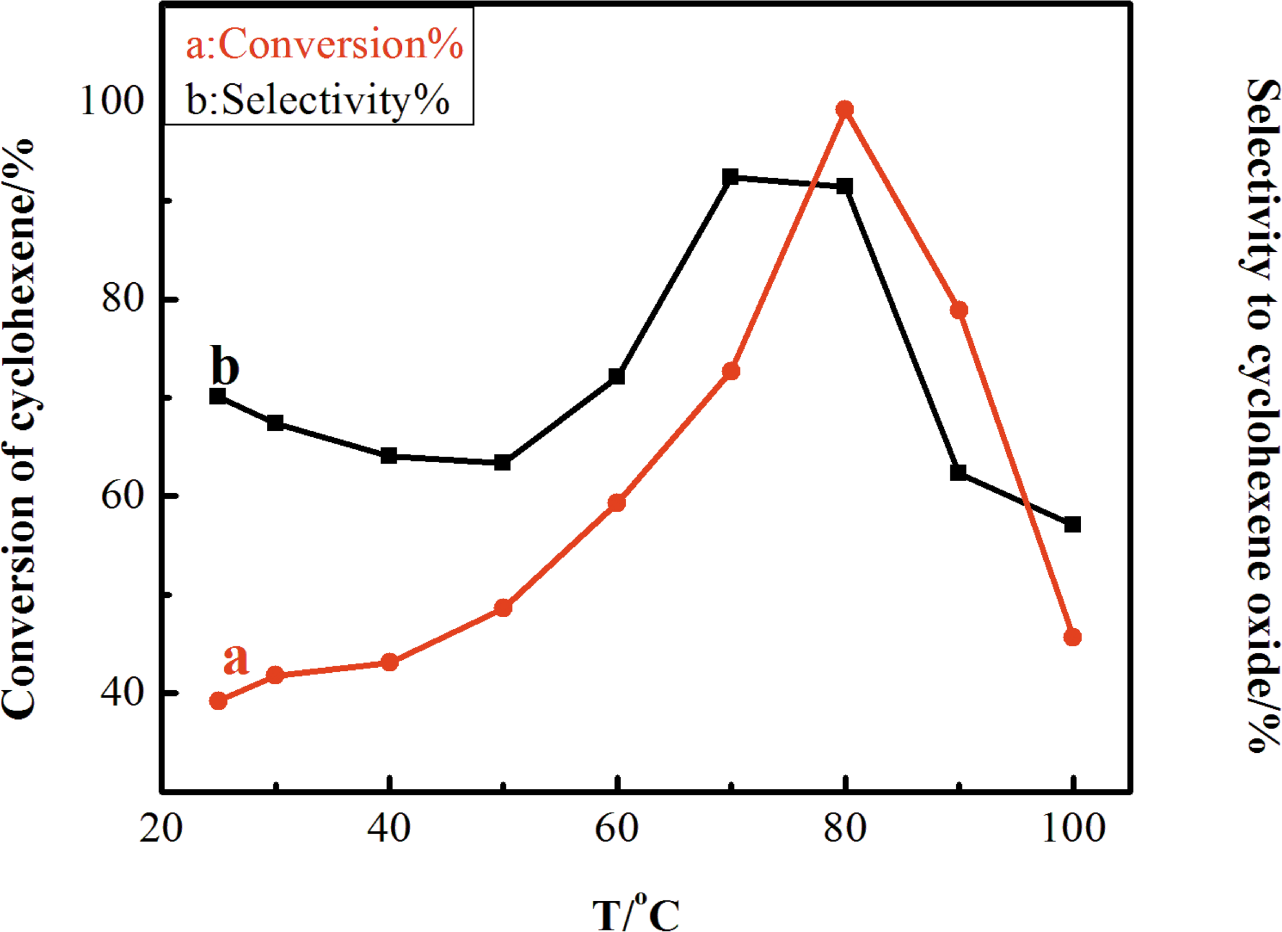
Effect of reaction temperature on the catalytic performance of Ag-TiO_2_-SiO_2_ in the epoxidation of cyclohexene. (Reaction conditions: catalyst, 80 mg; cyclohexene, 6 mL; H_2_O_2_, 6 mL; chloroform, 12 mL; for 120 min).

Fig 9 shows the effect of temperature on epoxidation reaction is investigated form 298 to 353 K. At 298 K, low conversion rate is achieved by the Ag-TS catalyst. With an increase in the reaction temperature, the cyclohexene conversion increases from 39.2 to 99.2% and then decreases obviously to 45.7% at 373 K. When the reaction temperature is over 353 K, the selectivity to cyclohexene oxide drops dramatically. It is clearly that increasing the reaction temperature can quicken the reaction rate, but the higher reaction temperature can accelerate the decomposition of H_2_O_2_. The oxidant is decomposed by high temperature before oxidation, resulting in the decrease in the cyclohexene conversion and the selectivity to cyclohexene oxide. The results in Fig 9 show that the heating process is necessary for the epoxidation of cyclohexene over Ag-TS catalyst, but this temperature is not too high. Thus, based on the obtained results, the optimum catalytic condition (best conversion and selectivity to cyclohexene oxide) is at 353 K.

**Fig 10:**
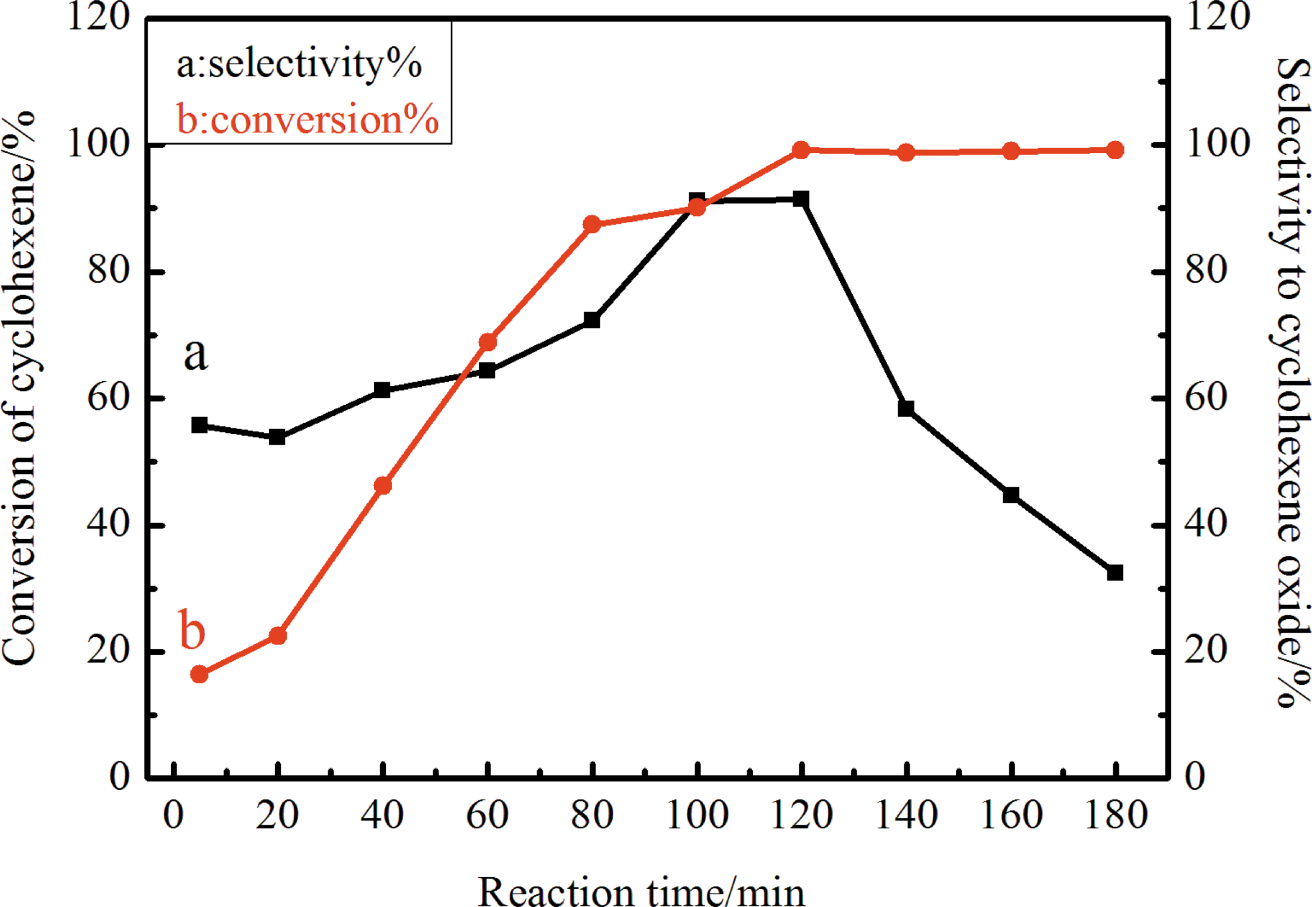
Effect of reaction time on the catalytic performance of Ag-TiO_2_-SiO_2_ in the epoxidation of cyclohexene. (Reaction conditions: catalyst, 80 mg; cyclohexene, 6 mL; H_2_O_2_, 6 mL; chloroform, 12 mL; at 353 K).

The conversion of cyclohexene and the product selectivity are monitored every 20 min in order to find out the optimum reaction time. Fig 10 shows the effect of the reaction time on the catalytic performance of Ag-TS in the epoxidation of cyclohexene at 353 K. With an increase in the reaction time from 5 to 180 min, the cyclohexene conversion increases from 16.5 to 99.2%, and the selectivity to cyclohexene oxide increases to a maximum for 120 min and then decreases obviously. When the reaction time is over 120 min, the cyclohexene oxide selectivity decreased from 91.4% to 32.4%, which was probably due to the subsequent reactions of the products. The result in Fig 10 shows that the reaction time is too long to proceed successfully to the target product. So it is very important to control the reaction time and the optimum reaction time for our system was 120 min.

**Fig 11:**
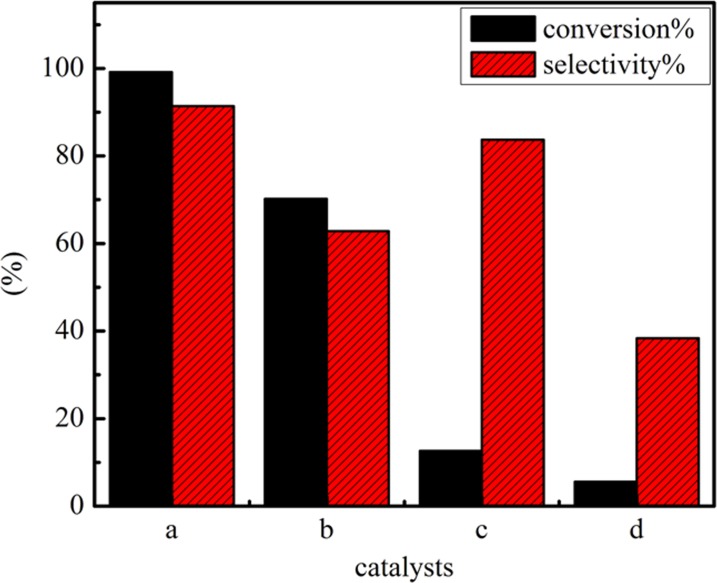
Effect on the catalytic performance of the three catalysts in the epoxidation of cyclohexene: (a) Ag-TiO_2_-SiO_2_; (b)Ag; (c) TiO_2_-SiO_2_; (d)blank (Reaction conditions: catalyst, 80 mg; cyclohexene, 6 mL; H_2_O_2_, 6 mL; chloroform, 12 mL; for 120 min).

The cyclohexene epoxidation is carried out using the three catalysts as oxidant and chloroform as solvent at 353 K for 120 K in Fig 11. TiO_2_-SiO_2_ particles show the poor catalytic ability. Using Ag as a catalyst, 70.2% cyclohexene oxide conversion is achieved, which indicates that Ag particles is able to oxidize cyclohexene. When Ag-TS composite are used as a catalyst, the higher conversion and selectivity of cyclohexene are obtained with the main product of cyclohexene oxide, 99.2% styrene oxide conversion and 91.4% selectivity are achieved. It is observed that the content of Ag in the catalysts played a crucial role in controlling the catalytic activity of the materials and the Ag species coated TiO_2_-SiO_2_ have a higher catalytic activity for the epoxidation of cyclohexene. As a matrix of Ag particles, TiO_2_-SiO_2_ particles possess higher activity for the epoxidation of cyclohexene and can provide more active sites for the adsorption of cyclohexene and therefore improve the cyclohexene conversion over Ag-TS catalyst. Our repeatable work is better than other similar researches [33–35], this demonstrates that TiO_2_-SiO_2_ in Ag-TS has led to a synergetic action. The presence of the TiO_2_-SiO_2_ composite oxide can enhance the reactants from contacting with the active sites using Ag-TS catalyst.

Table 1 shows the catalytic performance of Ag-TS composite calcined at different temperature for the epoxidation of cyclohexene. The results show that Ag-TS catalyst calcined at 723 K displays a higher cyclohexene epoxidation conversion and the most high epoxide yield. The conversion and selectivity of cyclohexene epoxidation are 99.2% and 91.4%, respectively. It is well-known that the transformation temperature of TiO_2_ particles form the amorphousness to crystals is in the range of 573–873 K. TiO_2_ of the TiO_2_-SiO_2_ particles was near completely transformed to the anatase phase at 723 K, but amorphous SiO_2_ were retained. The use of amorphous SiO_2_ support for the dispersion of titanium oxide offers many advantages such as large specific surface area, high thermal stability, and good mechanical strength [36]. Ag particles would grow during the calcination temperature, which also cause the particles agglomeration and reduce catalyst activity. On the other hand, the presence of a second phase could effectively hinder such growth. It is found that the optimal calcination temperature for the Ag-TS composite is 723 K.

**Table 1:** Effect of Ag-TS particle after calcination at different temperature on the catalytic performance in the epoxidation of cyclohexene at 353 K.

Catalyst (calcination temperature)	conversion (%)	product selectivity(%)			
		cyclohexene oxide	cyclohexenone	cyclohexenol	others
blank test	0.82	38.5	20.1	43.1	trace
Ag-TS(298 K)	38.4	50.8	13.8	15.7	19.7
Ag-TS(523 K)	56.3	42.8	19.9	31.6	5.7
Ag-TS(623 K)	77.4	20.2	19.4	6.6	53.8
Ag-TS(723 K)	99.2	91.4	4.1	3.8	0.7
Ag-TS(823 K)	99.0	48.6	13.6	29.3	8.5
Ag-TS(923 K)	95.7	15.7	57.2	8.0	19.1

(Reaction conditions: catalyst, 80 mg; cyclohexene, 6 mL; H_2_O_2_, 6 mL; chloroform, 12 mL; for 120 min).

Being important chemical intermediates in the fine chemicals industry, cyclohexene oxide, cyclohexenone, cyclohexenol and others are usually produced of by the selective epoxidation of cyclohexene with H_2_O_2_ as an oxidant. In the present research the epoxidation of cyclohexene is the primary product differs form the products of cyclohexene oxidization using other catalysts.

### Catalyst stability

**Fig 12:**
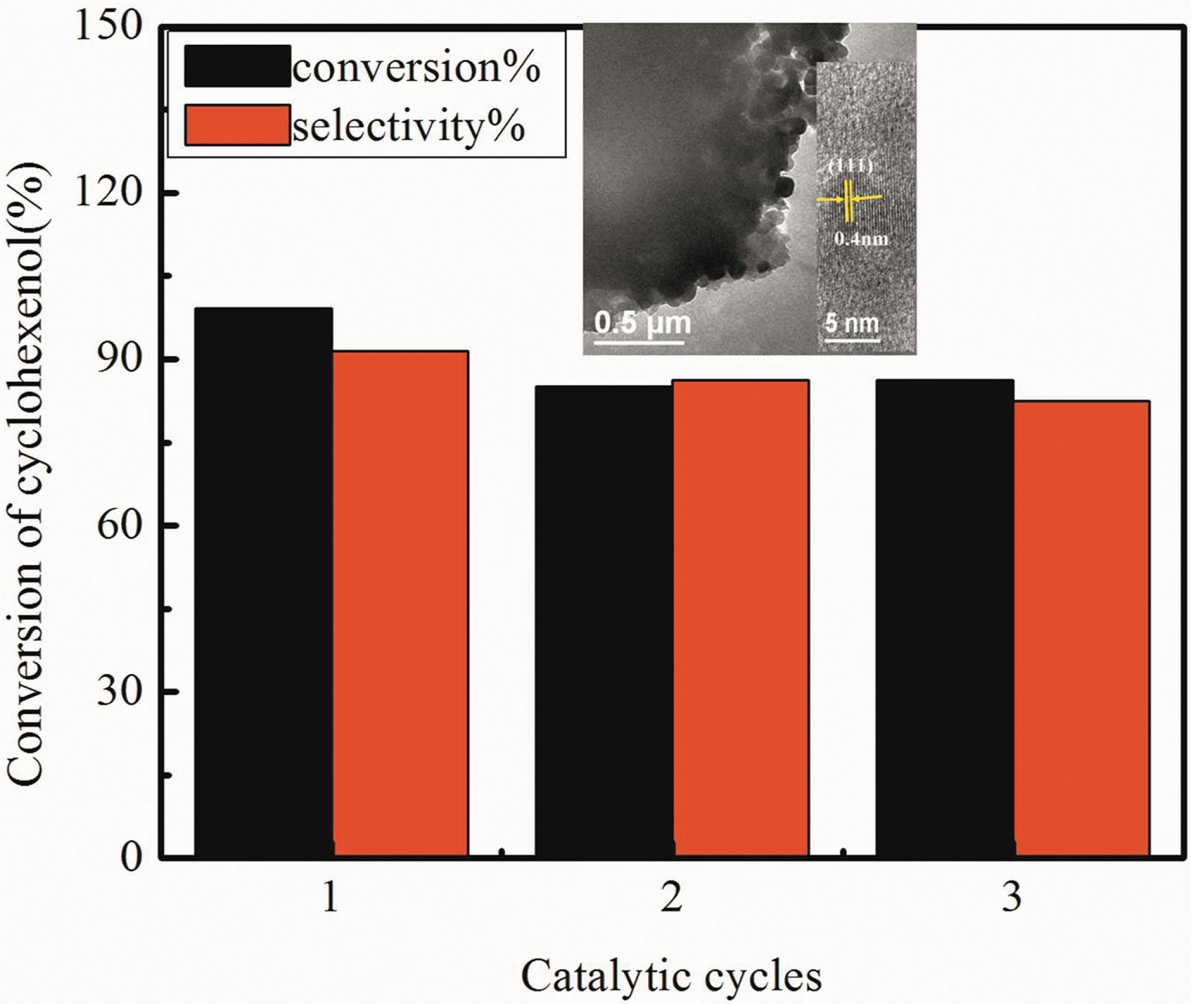
Catalytic stability of Ag-TS catalyst, the inset is TEM images after catalytic reaction of Ag-TS catalyst.

In order to examine the recyclability of the Ag-TS composite, the catalysts are recycled for three times to test their catalytic activities. After each cycle, the catalyst is recovered via centrifugation and washed with deionized water, dried at 353 K for 24 h and calcined in air at 723 K for 2 h. The reaction results show that the recovered Ag-TS catalyst after three recycles could maintain 83% of the initial catalytic activity(Fig 12). In the inset of Fig 12, the information about morphological structure of the synthesized catalyst after the catalytic reaction could be obtained by TEM. There was no significant change found in the morphology of catalyst. The HRTEM presents clear lattice fringe of the molecule packing, and the interlayer spacing is 0.4 nm which is corresponding to (111) facet of cubic crystal system.

## Conclusions

TiO_2_-SiO_2_ particles are fabricated on air-water interface via self-assembling process and used as the substrate of Ag particles. The Ag-TS composites are successfully prepared by a soaking method without employing any reducing agent. Experimental results show that Ag particles are tightly loaded on the surface of TiO_2_-SiO_2_. XRD, SEM, EDS, BET, TEM and UV-Vis have been used to characterize morphologies and structures of the catalyst. The catalytic activity of Ag-TS composite after calcination for the cyclohexene epoxidization depend on the noble metal(Ag) loaded on the surface of TiO_2_-SiO_2_. The product calcined at 723 K has the best catalytic activity. The TiO_2_-SiO_2_ particles possess synergistic catalytic activity. Using it as catalyst in cyclohexene oxidization reaction, the highest epoxide selectivity of 91.4% can be achieved. The reaction conditions, such as reaction time and reaction temperature have an obvious influence on the catalytic performance of Ag-TiO_2_-SiO_2_. In this experiment, the cyclohexene oxide is the major product. In addition, the activity of the Ag-TS catalyst remains nearly unchanged after three successive recycles of catalytic reactions. This indicates that the catalyst has a better stability of activity.
